# Simplest identification, O-specific polysaccharide purification and antigenic evaluation of Salmonella enterica serovar Typhi Vi negative isolate

**DOI:** 10.17179/excli2015-565

**Published:** 2015-10-07

**Authors:** Muhammad Salman, Aamir Ali, Abdul Jabbar, Yasra Sarwar, Moazur Rahman, Mazhar Iqbal, Abdul Haque

**Affiliations:** 1 Health Biotechnology Division, National Institute for Biotechnology and Genetic Engineering (NIBGE), Faisalabad, Pakistan affiliated with Pakistan Institute of Engineering and Applied Sciences (PIEAS), Islamabad, Pakistan; 2 Department of Biotechnology, Abdul Wali Khan University, Mardan; 3 Department of Biotechnology, Mirpur University of Science & Technology (MUST), Mirpur, AJK, Pakistan; 4 Department of Pathology, The University of Faisalabad, Faisalabad

**Keywords:** Salmonella Typhi Vi negative, O-Specific polysaccharides, typhoid vaccine

## Abstract

Currently licensed typhoid vaccines are based on Vi capsular polysaccharides. Recent molecular reports from typhoid endemic countries state that *Salmonella enterica *serovar Typhi (*S*. Typhi) Vi negative strains occur naturally and cause typhoid fever which is indistinguishable from disease caused by Vi positive strains. Vaccine based on Vi polysaccharide may not protect patients if the invading *S. *Typhi are negative for Vi. The lipopolysaccharide (LPS) is an essential component of *S. *Typhi outer membrane in which O-specific polysaccharide (OSP) is a protective antigen and universal candidate for vaccine development. In this study, *S. *Typhi Vi negative isolates were discriminated from Vi positive isolates through a duplex PCR using primers of *fliC-d* (599bp) and *tviA* (495bp) genes. The LPS of *S. *Typhi Vi negative isolates was extracted by hot phenol method and OSP was purified by core hydrolysis. The yield of extracted LPS was 91 mg/L and that of purified OSP was 49.14 mg/L of culture broth. LPS showed ladder like appearance by zinc imidazole staining following SDS-PAGE. Whole cell challenged mice sera were used for *in vitro* antigenicity evaluation of the purified LPS and OSP. The antigenicity was found adequate by immunodiffusion assay. To our knowledge, this is the first report of purification and antigenic evaluation of LPS of a Vi negative *S. *Typhi isolate. The purified OSP from *S. *Typhi Vi negative isolate may be coupled with a carrier protein to produce universal low cost conjugate vaccine candidates for use in typhoid endemic regions.

## Introduction

Generally typhoid fever is caused by *S. *Typhi Vi positive strains (STVP). However, *S. *Typhi Vi negative (STVN) strains which are emerging rapidly, can also contribute to this ailment (Baker et al., 2005[4]; Wain et al., 2005[17]; Pulickal et al., 2013[13]). In some Asian countries, particularly Nepal and Pakistan, STVN strains exist in nature frequently (Baker et al., 2005[4]; Pulickal et al., 2013[13]). Molecular studies have indicated that STVN isolates may not be agglutinated by anti-Vi sera (Arya, 2002[2]). These STVN isolates were identified during phage typing in various geographical locations as earlier as 1960s (Baker et al., 2005[4]). Even before these recent findings, a positive selection pressure on *S. *Typhi was observed, which down-regulated or stopped the expression of Vi subunit (Arya, 2002[2]). The expression of Vi capsule may not be crucial for infection in humans as STVN mutants and vaccine strains infect human volunteers and provoke immune response in them (Hone et al., 1988[8]; Pulickal et al., 2013[13]). These observations could have implications regarding the efficacy of the Vi based vaccine in typhoid endemic countries as well as for the conjugate vaccines based on Vi-proteins that are currently undergoing clinical trials for use in pediatric populations (Lin et al., 2001[9]; Pulickal et al., 2013[13]).

Lipopolysaccharide (LPS) is an essential structural component of the outer cell membrane of *Salmonella* species like other Gram negative bacteria. LPS plays a crucial role in pathogenesis in humans (Raetz and Whitfield, 2002[14]). OSP antigen is universally present both in Vi positive and Vi negative strains (Arya and Agarwal, 2014[3]). OSP of the LPS of *S. *Typhi is a protective antigen and has been used as a target for vaccine development (Ali et al., 2012[1]). 

Most of the previous work has been done on Vi positive *S*. Typhi isolates but this study was focused on purification and antigenic evaluation of OSP from a local Vi negative *S*. Typhi isolate.

## Materials and Methods

### Bacterial isolates and biochemical identification 

Two *S*. Typhi isolates (AS1 and AS7) were taken from National Institute for Biotechnology and Genetic Engineering (NIBGE) stock cultures, inoculated (50 µL) in 3 mL of tryptic soya broth (TSB, Merck, Germany) 30 g/liter (w/v) and incubated at 37 °C overnight. Subculture was done on a separate MacConkey agar plate (65 g/liter [w/v]) and incubated at 37 °C overnight. A single colony was transferred to triple sugar iron (TSI) agar slant to identify the *S. *Typhi. For biochemical identification of *S. *Typhi, Remel kit (RapID ONE) (Thermo Fisher Scientific, Lenexa, USA) was used according to the manufacturer's instructions. The results obtained were interpreted using software ERIC (Electronic RapID Compendium), (Thermo Fisher Scientific, Lenexa, USA).

### Molecular identification of S. Typhi Vi negative isolate

Total genomic DNA of biochemically identified *S. *Typhi was isolated as already described (Haque et al., 2001[6]). Duplex PCR was performed according to protocol mentioned earlier (Baker et al., 2005[4]). Two sets of primers: *fliC*-F/*fliC*-R was used for amplification of *fliC-d* gene and *Vi*-F/*Vi*-R (Table 1(Tab. 1), References in Table 1: Song et al., 1993[16]; Hashimoto et al., 1995[7]) was used to amplify *tviA* gene, present on *viaB* operon. The *fliC-d* gene was used as a signature sequence for the confirmation of *S*. Typhi while *tviA* gene was used to differentiate STVP and STVN isolates. 

### Fermentation of Vi negative S. Typhi

Confirmed STVN (AS7) isolate was grown in a 20 L fermenter (Biostat^®^C, USA) containing 12 L TSB and 10 % (v/v) inoculum. Fermentation was carried out at 32 °C and pH 6.7 with stirring at 400 rpm for 11 hours. After 7 hours, growth was supplemented with 25 % sterilized glucose solution to enhance the carbohydrate yield. Bacterial cells were killed using 1 % formalin. Stirring was continued at 200 rpm overnight and bacteria were harvested by centrifugation at 7,000 x g at 4 °C for 40 min.

### Purification of lipopolysaccharides (LPS) from S. Typhi

LPS was extracted from STVN by hot-phenol method as mentioned earlier (Westphal et al., 1965[18]). Briefly, the *S. *Typhi suspension (1 g of wet pellet per 10 mL of distilled water) was mixed with 90 % phenol (equilibrated at 68 °C) and incubated at the same temperature using inverted stirrer for 30 min. Temperature of the mixture was lowered to 10 °C using ice to enable the separation of phases followed by centrifugation at 7300 x g for 50 min at 10 °C. The upper clear aqueous layer was collected. The amount of water (equal to the collected water layer) was added to the lower layer and the mixture was again stirred at 68 °C for 30 min, cooled to 10 °C and centrifuged at 7300 x g for 50 min. The two water layers were combined and re-centrifuged at 8000 x g for 50 min. The upper layer was collected and precipitated contaminants were discarded. The water layer was adjusted to contain 10 mM sodium acetate, 2 mM calcium chloride and 25 % ethanol, and incubated at 4 °C overnight. The suspension was centrifuged at 14,300 x g at 10 °C for 60 min to precipitate the nucleic acids. To precipitate the LPS, 75 % ethanol was added to supernatant, mixed at room temperature for 20 min and kept at 4 °C overnight. The supernatant was removed by centrifugation at 7,300 x g for 60 min at 4 °C.The pellet was dissolved in appropriate amount of deionized distilled water and dialyzed (cut off 6,000 - 8,000) for 2 days against deionized distilled water with 2 water changes per day. The dialyzed pellet was lyophilized and named as crude LPS. The crude LPS were dissolved in deionized distilled water, dispensed into a dialysis tube and equilibrated against buffer (2 mM MgSO_4_, 50 mMTris-Cl, pH 7.6) at 37 °C for 2 hours. After equilibration, DNase (200 µg/mL) and RNase (50 µg/mL) were added whereas proteinase K (200 µg/mL) was added after 6 hours. The mixture was further dialysed overnight at 37 °C. Next day, the dialysis buffer was replaced with deionized water and dialysis was continued at 4 °C for 2 days with 2 water changes per day. The dialyzed suspension was centrifuged at 5500 rpm for 30 min, pellet was discarded and the supernatant was ultracentrifuged at 96,000 x g for 5 hours at 4 °C. The pellet was lyophilized as purified LPS. For nucleic acid estimation, the LPS was dissolved in 1 % sodium dodecyl sulfide solution (1 mg/mL) and scanned at 260 nm using 1 % SDS as blank solution. The nucleic acids concentration was analyzed.

### Purification of O-specific polysaccharides

Core hydrolysis of the purified LPS was done for OSP extraction as described earlier (Ali et al., 2012[1]; Chu et al., 1991[5]). Briefly, the purified LPS were dissolved at 10 mg/ mL in 1 % glacial acetic acid and placed in a reaction-block at 100 °C for 90 min. The mixture was cooled to ambient temperature; distributed in Corex tubes and centrifuged at 8000 x g for 20 min. Supernatant was passed through a chromatography column using G-25 Sephadex (Amersham Biosciences, USA). The void volume fractions were pooled in a round bottom flask, frozen with dry ice and lyophilized. The nucleic acid and protein concentration was analyzed as mentioned earlier (Chu et al., 1991[5]). The purified OSP was stored at -20 °C.

### Immunodiffusion assay of purified LPS and OSP

One percent agarose gel was used for immunodiffusion assay (Ouchterlony, 1986[11]) and the assay was performed on glass slides. Hyper immune sera were raised by injecting mice for 3 consecutive weeks with 3 injections per week of the formalin killed *S*. Typhi whole cells (Ali et al., 2012[1]). Heart bleeding was done after one week of the last injection and the serum was separated and used for the immunodiffussion assay.

## Results

### Biochemical identification of S. Typhi

AS1 and AS7 showed smooth, transparent, round (2-3 mm) and moist colonies on MacConkey agar plates. On TSI agar slants, the *S. *Typhi isolates produced yellow pigment in butt (due to acid production) and showed pink slant surface (due to alkali production). Small amount of blackening of the medium was observed due to H_2_S production. However, no gas production was recorded. These typical biochemical reactions as well as characteristic reactions on Remel kit (Thermo Fisher Scientific, Lenexa, USA) observations identified both of the isolates as *S*. Typhi.

### Duplex PCR for confirmation of Vi negative S. Typhi isolate

**Figure 1:**
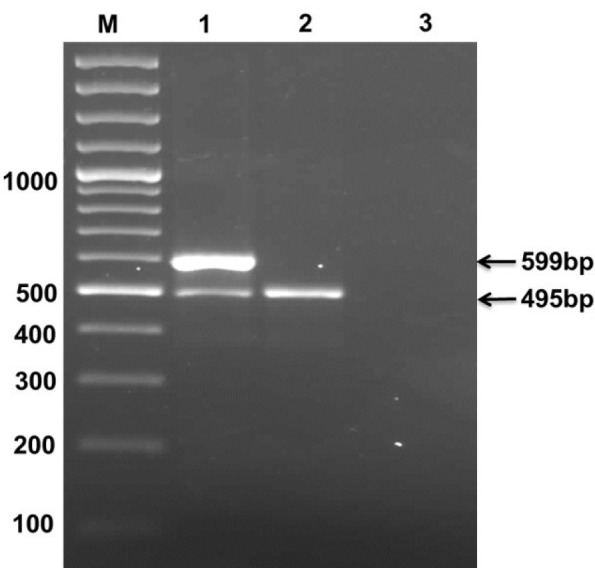
S. Typhi genomic DNA of Vi positive and Vi negative isolates were amplified using duplex PCR Lane 1; primers of *fliC* and *Vi* genes were amplifed from Vi positive isolate, Lane 2; only *fliC* gene was amplified from Vi negative isolate. Lane 3; negative control that did not show any result. Lane M denotes 100 bp DNA ladder (Fermentas Cat No. SM323).

Duplex PCR resulted in amplified products of both *fliC-d* (495 bp) and *tviA* (599 bp) gene fragments for STVP isolate (AS1), while for STVN (AS7), amplification of only *fliC-d* (495 bp) gene fragment was seen. No PCR amplification was found in case of negative controls. This molecular analysis confirmed AS7 as STVN isolate (Figure 1(Fig. 1)).

### Purification of lipopolysaccharides (LPS) from S. Typhi

**Figure 2:**
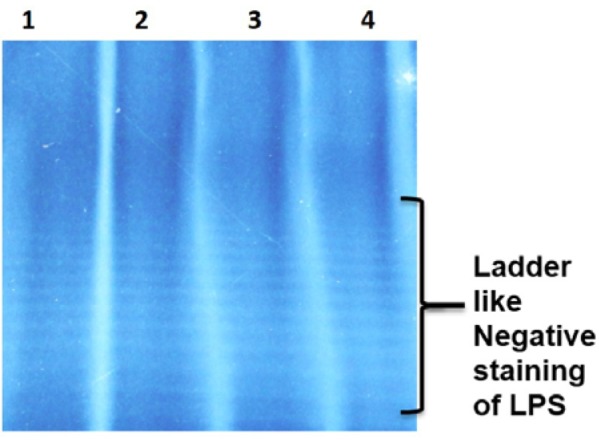
Lane 1-4 contain 2, 1, 0.5 and 0.25 mg/mL of S. Typhi Vi negative LPS respectively followed by Zinc imidazole stain. Due to repetitive sugars concentration the LPS shows ladder like structure instead of a single band.

STVN AS7 isolate when grown in fermenter, yielded 18.18 g of wet pellet per liter of culture. LPS were extracted and purified from the cell pellet at a concentration of 91 mg of purified LPS per liter culture. In crude LPS, nucleic acid contamination was found as 9.38 % while the protein impurities were detected to be 9.78 %. The nucleic acid and protein impurities were reduced to 0.06 % and 0.07 % respectively by treatment of DNase, RNase and protease (Table 2(Tab. 2)). The purified LPS were checked on SDS-PAGE followed by zinc-imidazole staining, which showed characteristic multiple repetitive band pattern (Figure 2(Fig. 2)).

### Purification of O-specific polysaccharides (OSP) of S. Typhi

**Figure 3:**
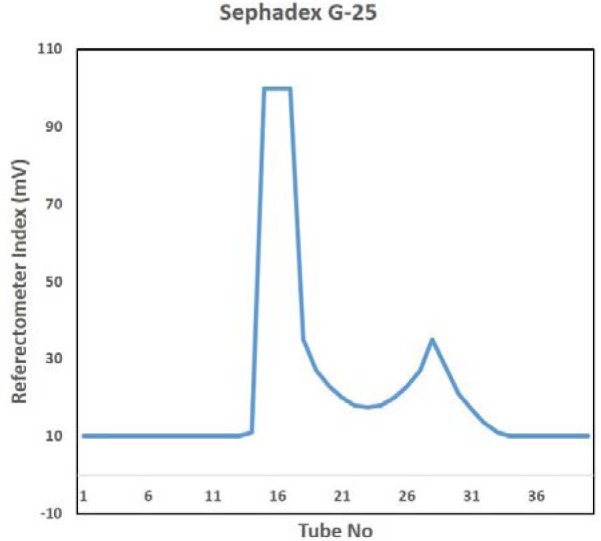
Sephadex G-25 size exclusion chromatography column for purification of O-specific polysaccharide (OSP) of S. Typhi Vi negative using phosphate buffer saline (PBS) citrate as mobile phase. X-axis shows tube number and Y-axis denotes refractive index.

Sephadex G-25 size exclusion chromatography of core hydrolyzed LPS resulted in lipid A removal and yielded the purified OSP as 49.14 mg/L yield of OSP (54 % OSP from purified LPS) (Figure 3(Fig. 3)). The nucleic acid and protein contaminations were found as 0.04 % and 0.03 % respectively (Table 2(Tab. 2)).

### Antigenicity evaluation of purified LPS and OSP

**Figure 4:**
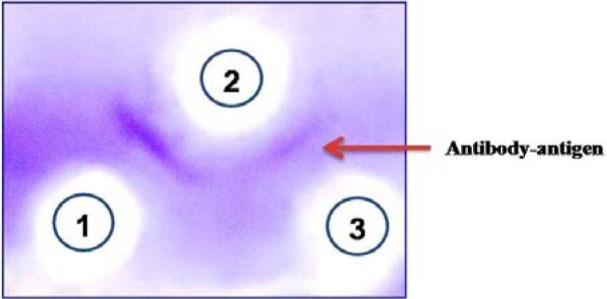
To determine the antigen antibody interaction *in vitro*, LPS of S. Typhi Vi negative and mice serum (polyclonal antibodies) were loaded on 1 % agarose immunodiffusion gel. Well 1 and 3 shows 1 mg/mL and 0.5 mg/mL LPS in 1 N saline respectively, while well 2 shows 1/8 time diluted polyclonal antibodies.

The immunodiffusion assay showed a clear precipitin line between antigens (LPS/ OSP) and antibodies (Figure 4(Fig. 4)) which confirmed that LPS/OSP were antigenically active and can further be used for conjugation with carrier protein to make potential immunogenic conjugate vaccine candidates.

## Discussion

Vi capsular polysaccharide of *S. *Typhi is encoded on a region of the bacterial genome called *viaB* operon which is located on the *Salmonella* Pathogenicity Island-7 (SPI-7) that consists of 10 genes: 5 coding for the synthesis of the polysaccharide capsule (*tviA, tviB, tviC, tviD, tviE*) and 5 coding for the polysaccharide transportation proteins (*vexA, vexB, vexC, vexD, vexE*) (Pickard et al., 2003[12]). Earlier studies have confirmed the expression of Vi capsular polysaccharide gene. On these basis, it is easy to confirm STVN isolates (Maurya et al., 2010[10]).

Generally, it is assumed that the onset of typhoid fever in humans is because of Vi antigen (Wetter et al., 2012[19]). On the contrary, typhoid fever may be caused by naturally occurring STVN strains having similar symptoms as caused by STVP strains. In a study from Faisalabad, Pakistan, 60 fresh isolates from typhoid patients' blood were examined and 9 (15 %) were found negative for both *tviA* and *tviB* genes (Baker et al., 2005[4]). In India, 10 % of freshly isolated strains were also found to be negative for Vi agglutination (Maurya et al., 2010[10]). Very recently the prevalence of STVN isolates was confirmed in children from Kathmandu, Nepal. Out of sixty eight isolates, 5.9 % of total isolates were found negative for capsular expression through slide agglutination tests (Pulickal et al., 2013[13]). Suppression of Vi expression enhances the capability of *S. *Typhi invasion in the intestine and facilitate the destruction within Peyer's patches (Zhao et al., 2001[20]). There are few reports for differentiating STVP and STVN isolates but they need use of a large number of primers. This method is a simple duplex PCR.

Vaccine based on Vi polysaccharide will not protect patients if the invader *S. *Typhi is negative for Vi (Senthilkumar et al., 2014[15]). So it is imperative to target antigens which are universally present in all *S. *Typhi. Lipopolysaccharides (LPS) of outer cell membrane have been identified as having potential for development of vaccines effective against all *S. *Typhi isolates. In LPS, Lipid A is a toxic constituent, while OSP chains play an important role in resistance to serum bactericidal action and phagocytosis (Raetz and Whitfield, 2002[14]). It is important to remove Lipid A. To date, there is no report of extraction of LPS and purification of OSP from STVN isolates. During this study, LPS was extracted from STVN by using the Westphal method. Impurities of nucleic acid and proteins from the extracted LPS were reduced up to 99 %. Furthermore, passing the purified OSP through Sephadex G-25 removed the residues of enzymes. The obtained yield of purified OSP was 49.14 mg/L (Table 2(Tab. 2)).

In the immunodiffusion reaction, clear precipitin lines were obtained using LPS/ OSP concentrations of 1 mg/mL and up to a 1/8 dilution of the mice serum containing IgG antibodies. No positive reactions were observed with normal saline control.

This is the first report of purification and antigenic evaluation of OSP from STVN. The purified OSP can be used in preparation of universal vaccine candidates after conjugation with a carrier protein. 

## Notes

Muhammad Salman (Department of Biotechnology, Abdul Wali Khan University, Mardan, Cell: +92 3349721666; E-mail: msalman@awkum.edu.pk; s.amazai@yahoo.com) has contributed as co-corresponding author.

## Acknowledgements

We are thankful to National Institute for Biotechnology and Genetic Engineering (NIBGE), Faisalabad, Pakistan for providing research facilities and grateful to Higher Education Commission (HEC), Pakistan as funding source for this work.

## Declaration of interest

The authors report no conflicts of interest. 

## Figures and Tables

**Table 1:**
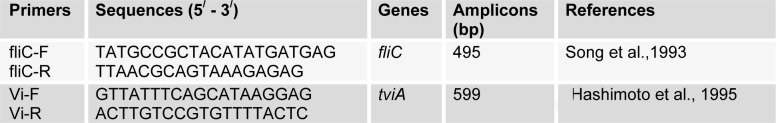
Primers used in duplex PCR for Vi negative S. Typhi identification

**Table 2:**
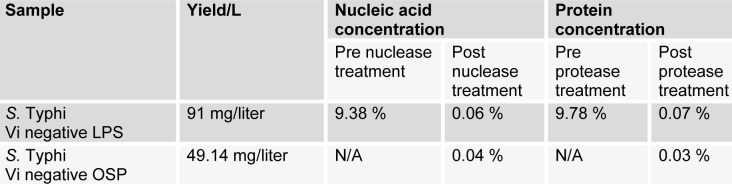
Nucleic acid and protein concentration in Vi negative S. Typhi LPS
